# Systematic CT evaluation of reduction and hardware positioning of surgically treated calcaneal fractures: a reliability analysis

**DOI:** 10.1007/s00402-017-2744-5

**Published:** 2017-07-26

**Authors:** R. J. O. de Muinck Keizer, M. S. H. Beerekamp, D. T. Ubbink, L. F. M. Beenen, T. Schepers, J. C. Goslings

**Affiliations:** 1 Trauma Unit, G4-137, Department of Surgery, Academic Medical Center, PO-box 22660, 1100 DD Amsterdam, The Netherlands; 2 Department of Surgery, Academic Medical Center, Amsterdam, The Netherlands; 3 Department of Radiology, Academic Medical Center, Amsterdam, The Netherlands

**Keywords:** Calcaneus, Fracture, Surgical treatment, Computed tomography, Evaluation

## Abstract

**Introduction:**

Up to date, there is a lack of reliable protocols that systematically evaluate the quality of reduction and hardware positioning of surgically treated calcaneal fractures. Based on international consensus, we previously introduced a 23-item scoring protocol evaluating the reduction and hardware positioning in these fractures based on postoperative computed tomography. The current study is a reliability analysis of the described scoring protocol.

**Methods:**

Three raters independently and systematically evaluated anonymized postoperative CT scans of 102 surgically treated calcaneal fractures. A selection of 25 patients was scored twice by all individual raters to calculate intra-rater reliability. The scoring protocol consisted of 23 items addressing quality of reduction and hardware positioning. Each of these four-option questions was answered as: ‘optimal’, ‘suboptimal (but not needing revision)’, ‘not acceptable (needing revision)’ or ‘not judgeable’. We used intraclass correlation coefficients (ICC’s) to calculate inter- and intra-rater reliability.

**Results:**

Inter-rater reliability of the overall 23-item protocol was good (ICC 0.66, 95% CI 0.64–0.69). Individual items that scored an inter-rater ICC ≥0.60 included evaluation of the calcaneocuboid joint, the posterior talocalcaneal joint, the anterior talocalcaneal joint, the position of the plate and sustentaculum screws and screws protruding the tuber and medial wall. The intra-rater reliability for the overall protocol was good for all three individual raters with ICC’s between 0.60 and 0.70.

**Conclusion:**

Our scoring protocol for the radiological evaluation of operatively treated calcaneal fractures is reliable in terms of inter- and intra-rater reliability.

## Introduction

The main goal of surgical treatment of calcaneal fractures is to restore the anatomy, as intra-articular incongruences are associated with posttraumatic osteoarthritis of the subtalar joint and poor clinical outcomes [1–3]. To adequately restore the anatomy, different surgical techniques have been proposed [4]. To compare the radiological results of these techniques, a blinded, independent radiological assessment with a fixed set of reliable criteria should be standard.

Unfortunately, there is lack of a validated scoring protocol on the qualitative assessment of calcaneal fracture reduction and hardware positioning [5–10]. As evaluation of plain radiography seems insufficient [11], different computed tomography (CT) based measurements have been proposed [12, 13]. Individual studies use different thresholds to specify acceptability of angles or intra-articular congruity [8, 11, 13–16]. Additionally, reliability of these measurements is only seldom reported.

A recently published international Delphi consensus on how to evaluate postoperative results of surgically treated calcaneal fractures showed that in addition to the quality of reduction, the quality of hardware positioning also requires evaluation [17]. Additionally, it showed that measurements were performed scarcely in clinical practice; evaluation of both reduction and hardware positioning is mostly performed by expert opinion.

Based on this international consensus, a fixed set of criteria for the assessment of the quality of fracture reduction and hardware positioning of the calcaneus has been composed. The aim of the current study was to determine the inter- and intra-rater reliability of this radiological scoring protocol.

## Methods

To determine the inter- and intra-rater reliability of the scoring protocol, we used postoperative CT scans of 100 patients with 102 surgically treated calcaneal fractures. These patients had been enrolled in the EF3X-trial, a multicenter randomized clinical trial exploring the clinical value of additional 3D fluoroscopic imaging in the treatment of calcaneal fractures [18].

Postoperative CT scans were anonymized and systematically evaluated with use of the scoring protocol by three independent raters [an experienced foot- and ankle surgeon (TS), a radiologist with specialty in musculoskeletal trauma (LFB), and a surgical trainee in orthopaedic surgery and PhD candidate with 4 years of research experience in calcaneal fractures (RJDMK)]. No three-dimensional (volume rendering) reconstructions were available.

The scoring protocol used was developed after Delphi consensus between 18 international experts in the field (both surgeons and radiologists) and previously published in this journal [17]. The protocol consists of 23 items addressing post-operative reduction and hardware positioning of the most important anatomical landmarks of the calcaneus (Table 2). Each of these multiple-choice questions was answered as: ‘optimal’, ‘suboptimal (but acceptable)’, ‘not acceptable (revision required)’ or ‘not judgeable’. In case of gaps and steps a threshold of 2 mm was held for acceptability [19]. After scoring 23 items separately, a concluding dichotomous question was answered about whether any of the findings required correction (i.e. Yes or No). Statistical analyses were performed with SPSS (IBM SPSS Statistics for Windows, Version 22.0. Armonk, NY, USA).

### Inter-rater reliability

We used a two-way random, average measures, absolute agreement intraclass correlation coefficient (ICC) to determine the degree of agreement amongst raters, including its 95% confidence interval (CI). As we used a fully crossed design (all subjects were rated by all raters) we chose a two-way model [20]. As we intended to generalize the results to a larger population of clinicians, we chose a random effects model [21]. A good inter-rater reliability (IRR) was characterized by absolute agreement and not by consistency in the ratings. Concerning interpretation, we expect the protocol to be used in a clinical research environment were postoperative results are scored by more than one rater. Consequently, we primarily calculated the average-measures ICC. We used cutoffs as provided by Cicchetti et al., with reliability being ‘poor’ for ICC values less than 0.40, ‘fair’ for values between 0.40 and 0.59, ‘good’ for values between 0.60 and 0.74, and ‘excellent’ for values between 0.75 and 1.0 [22]. An ICC ≥0.60 was set as minimally acceptable level of agreement [22].

### Intra-rater reliability

After a minimum of 30 days of scoring, raters were asked to again evaluate a selected subset of 25 CT scans that they had seen before but had been given a new study ID. These cases were selected to represent the full range of postoperative results, i.e. from anatomical reduction and correct screw positioning to large intra-articular step-offs, malreduced Böhler angles and intra-articular screws—and everything in between. Scoring results of both sessions were combined in a database per rater to analyze the degree of agreement within the observations (i.e. intra-rater reliability). In contrast to the inter-rater reliability, we used a two-way mixed, absolute agreement, single measures ICC as we wanted to determine the degree of agreement with the raters own ratings and do not intend to extrapolate this to a different rater [21]. As for the inter-rater reliability, a good reliability was characterized by absolute agreement and not by consistency in the ratings. Again, cutoffs were used as provided by Cicchetti et al. [22].

## Results

The inter-rater reliability of the overall 23-item protocol was good: ICC of 0.66 (95% CI 0.64–0.69) (Table 1). Individual items that scored an inter-rater ICC ≥0.60 included the calcaneocuboid (CC) joint (symmetry/width, intra-articular steps, gaps and screws), the posterior talocalcaneal (PTC) joint (symmetry/width, intra-articular steps, gaps and screws), the anterior talocalcaneal (ATC) joint (intra-articular screws), the position of the plate and the sustentaculum screws and screws protruding the tuber and medial wall. Items that did not score acceptable inter-rater agreement (ICC < 0.60) included Böhler’s and Gissane’s angles, length of the calcaneus and varus/valgus position of the tuber, intra-articular fragments in CC, PTC or ATC joints, intra-articular gaps and step-offs in the ATC and the positioning of anterior process screws. When only the items that scored an acceptable ICC (≥0.60) were combined, the protocol scored 14 items (Table 1, marked grey) and had an excellent overall inter-rater reliability with an ICC of 0.77.

**Table 1:**
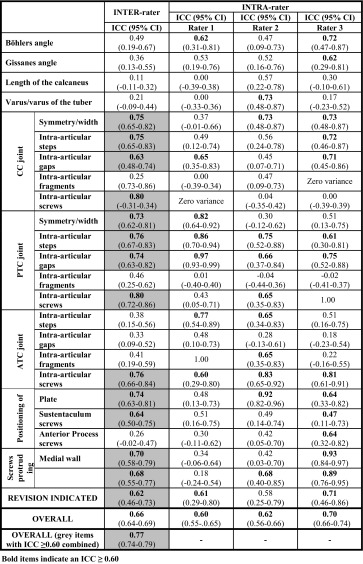
Inter- and intra-rater reliability per item.

The intra-rater reliability for the overall protocol was good for all three individual raters with ICC’s between 0.60 and 0.70. Individual raters scored acceptable ICC’s for an average of 11 items. Items that scored an ICC ≥ 0.60 for all three raters included steps and gaps in the PTC joint and presence of intra-articular screws in the ATC joint. Items that did not score acceptable ICC’s with any of the raters included length of the calcaneus, intra-articular fragments and screws in the CC joint, fragments in the PTC joint and gaps in the ATC joint.

## Discussion

Our scoring protocol assessed quality of both reduction and hardware positioning and showed a good inter-rater reliability based on 300+ observations, suggesting sufficient reliability for use in clinical and research settings. It can aid future studies in the structural comparison of treatment results in the field of operatively treated calcaneal fractures, where there is currently no practicable alternative.

Calcaneal fractures are often complex and classification systems typically show poor to moderate inter-rater reliability [23]. Scoring protocols on the postoperative evaluation of these fractures are numerous, but often do not mention data on reliability or only focus on (parts of) fracture reduction.

In 2003, Gupta et al. used pre- and postoperative CT scans to measure 7 displacement parameters in 32 calcaneal fractures. Measurements were done by a single rater without providing intra-rater reliability [12]. Sahota et al. focused on the postoperative alignment of the posterior facet [24]. They reported excellent inter-rater reliability between three independent raters by comparing ten postoperative CT scans. Kurozumi et al. evaluated parameters of calcaneal deformity by comparing postoperative CT images of both the injured and healthy contralateral side [13]. They found better reduction of the posterior facet and better reduction of the calcaneocuboid joint to be prognostic factors of functional outcome, but did not provide data on reliability of their measurements. In 2010, Magnan et al. performed postoperative CT analysis of 54 patients with calcaneal fractures using the Score Analysis of Verona (SAVE) [4, 25]. The SAVE scoring system was specifically designed for CT evaluation of calcaneal fractures and describes five displacement parameters [4, 25]. After a mean follow-up of 49 months, parts of the score showed statistical correlation with the clinical outcome as judged by the Maryland Foot Score: better clinical outcomes showed a significant association with vertical/longitudinal realignment and restoration of the calcaneal height [25]. Despite its correlation with clinical outcome, data on the reliability of the SAVE scoring system is currently unavailable. Finally, in 2014, Sanders et al. described a long term follow-up of 108 surgically treated patients with his well-known Sanders classification [26]. In addition to his traditional fracture classification [27], he added measurements of posterior facet congruity, dividing the extent of anatomic reduction in four categories. They confirmed that after 10–20 years of follow-up, the classification was still prognostic for outcome, as worsening outcome occurred with higher Sanders fracture types. However, included patients only had one of two types (Sanders II vs Sanders III). No data on reliability were published.

Although all abovementioned scoring systems were specifically designed for post-operative evaluation, none of them assessed hardware positioning such as presence of intra-articular or medially protruding screws.

We have chosen to base this scoring protocol on CT imaging as it is currently the golden standard with respect to the visualization of intra-articular gaps, step-offs and hardware positioning [13]. Nonetheless, despite its qualities, some measurements might be poorly visible on CT imaging. Böhler’s and Gissane’s angle measurements were originally designed for lateral radiographs. We hypothesized that estimation of these angles could be done by scrolling through the sagittal reconstructions of the CT scan. In addition, as mentioned by Kurozumi et al., Böhler’s angle comprises multiple factors: anterior lateral wall, PTC, and tuber displacement: all of which are evaluated separately with CT imaging [13]. Still, in line with the existing literature, we did not produce high reliability of Böhler’s and Gissane’s angle measurements on CT [23, 28].

The posterior talocalcaneal (PTC) is widely regarded as having the largest impact on post-operative complaints [29–32]. In contrast to measurements of Böhler’s angle, measurements of the PTC joint scored good agreement on four out of five items. The presence or absence of intra-articular bone fragments scored only fair agreement, possibly due to disagreement with regard to the posterior limits of the PTC joint.

On a statistical note, reliability analyses are frequently reported by the percentage that raters agree in their ratings, often referred to as percentage agreement. However, this measure systematically overestimates the level of agreement by not correcting for agreement that would be expected by chance alone [20]. The intraclass correlation or ICC is a measure that is suitable for ordinal, interval and ratio variables. It incorporates the magnitude of disagreement as does a weighted kappa, but has the advantage that it can handle more than two raters [33].

To accurately calculate inter-rater reliability, sufficient variance in the observed cohort is indispensable. For instance, very low prevalence of intra-articular screws in the CC joint can cause a low ICC. The low variance for this item is expressed by a broad range of the 95% confidence interval, suggesting a low representability of the ICC.

Some items have a high inter-rater (>0.6) but a low (<0.6) intra-rater reliability within individual raters. Raters can agree with each other at a certain moment, but not with themselves the next. This variability is inherent to classification systems, and in our case, does not hamper the good overall reliability of the scoring protocol.

Instead of exact measurements that are mostly performed in research settings, we have used subjective evaluations (e.g. good, moderate or poor). Subjective evaluation dismisses the need for tedious measurements, thereby allowing for a broader, more extensive evaluation without extending the burden of the task. In addition, subjective (categorical) and objective (numerical values) evaluations have previously proven to have a good correlation [34]. Moreover, during surgery no measurements can be performed and all the surgeon can do is estimate the quality of reduction and fixation, based on his experience with the acceptable angle measurements and distances.

This is also where a potential underestimation of the inter-rater reliability comes in: we used raters with sufficient expertise, but a different background. A radiologists’ perspective is likely to be different to that of a foot and ankle surgeon, especially when asked for a subjective opinion; e.g. the term “acceptable” could have different meanings for the two based on (a lack of) surgical experience. Undoubtedly, inter-rater reliability suffers from this phenomenon and is expected to be higher when rating is performed solely by experienced foot and ankle surgeons.

In the original study published in this journal we concluded that more items required evaluation than traditionally used in scoring protocols [17]. However, the current study shows that many of the 23 items scored do not show sufficient inter-rater reliability. If we would design a protocol using only the items that scored an inter-rater reliability of 0.6 or higher, this protocol would evaluate 14 items and have an excellent reliability with an ICC of 0.77. This would, however, discard the previously mentioned consensus and potentially ignore items with high predictive value of functional outcome. Future studies should focus on identifying which items indeed correlate with functional outcome to help optimize the reliability and usability of the current protocol.

In conclusion, the results of the present study show that our previously developed scoring protocol for the radiological evaluation of operatively treated calcaneal fractures is reliable in regard to inter- and intra-rater reliability. The scoring protocol can be used in future clinical research settings that focus on the radiological comparison of operatively treated fractures of the calcaneus.

## Figures and Tables

**Table 2:**
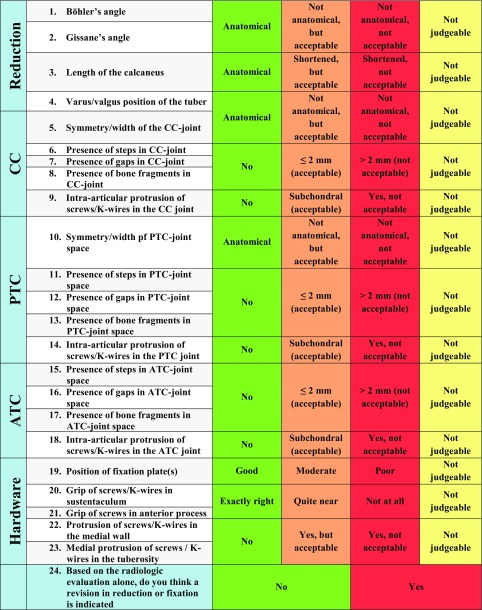
The scoring protocol as based on international Delphi consensus

## Appendix

See Table 2.
